# Risk of fracture according to glucocorticoid use after renal biopsy: a nationwide population-based study

**DOI:** 10.1038/s41598-020-70935-w

**Published:** 2020-08-14

**Authors:** Eunyoung Lee, Min-Jeong Lee, Bumhee Park, Inwhee Park

**Affiliations:** 1 Department of Biomedical Informatics, Ajou University School of Medicine, Suwon, Republic of Korea; 2 Office of Biostatistics, Ajou Research Institute for Innovative Medicine, Ajou University Medical Center, Suwon, Republic of Korea; 3 Department of Nephrology, Ajou University School of Medicine, Suwon, Republic of Korea

**Keywords:** Nephrology, Risk factors

## Abstract

Few data are available regarding fracture risk in patients treated with glucocorticoids, including patients with kidney disease. A population-based retrospective cohort study was performed using Health Insurance Review and Assessment Service database, a South Korean nationwide cohort set. This study identified 44,702 patients with diagnosis code of kidney diseases who received a renal biopsy between January 1, 2012 and December 31, 2017. A total of 8,624 patients met all study inclusion criteria. A total of 1,406 fractures of any site were observed in the study period. The glucocorticoid-exposed group had more fractures than the unexposed (14.4% vs 8.8%, P < 0.0001). Vertebral fractures were the most common, followed by upper limb, and lower limb fractures. The exposed group showed a remarkably higher hazard ratio of fracture risk (HR 6.0, 95% CI 5.01–7.23) than the unexposed group, indicating systemic glucocorticoid exposure was highly associated with fracture risk. Although HR increased at doses even less than 5 mg/day, it was independent of dose. Older age showed a significant effect on fracture risk (HR 1.2, 95% CI 1.05–1.44), even after adjusting for systemic glucocorticoid exposure. Glucocorticoids was associated with higher risk of fracture even at a low daily dose and short term exposure.

## Introduction

Glucocorticoids have been extensively used in the treatment of immune-related kidney diseases because of their anti-inflammatory and immunosuppressive effects^1^. However, the use of systemic glucocorticoids is associated with a range of adverse effects, including increased risks of osteoporosis and fracture^2^. These agents can decrease the net absorption of calcium and inhibit bone formation^3^. Glucocorticoid-induced osteoporosis and osteopenia have been reported in as many as 30–50% of patients receiving chronic glucocorticoid therapy^3,4^. Many studies using population-based cohort of adults have documented increased fracture risk of rheumatoid arthritis (RA), pulmonary disease, and inflammatory bowel disease after glucocorticoid exposure^2,5^. However, data on the risk of glucocorticoid-associated fractures in patients with kidney disease are lacking. Assessing the relation between glucocorticoid use and fracture risk is challenging because exposures are dynamic and difficult to measure. In addition, the association may be confounded by independent associations of fracture risk with underlying diseases, patient age, and sex. Despite the same use of glucocorticoid, kidney disease and other diseases differ in several underlying conditions. The dosage and the duration of the use of glucocorticoid for kidney disease are different from those for other diseases. They tend to be started with pulsating and finished with shorter or longer duration for kidney disease than for other chronic inflammatory diseases^6,7^. The sex and age at which kidney disease occurs are also different from those of other diseases^8^. Focusing on patients who received a kidney biopsy, the objective of this study was to determine potential independent effects of glucocorticoids on fracture risk. It may provide evidence for quantifying a relation between glucocorticoid use and fracture risk in kidney disease. In this study, we compared the incidence and fracture risk between patients with and without glucocorticoid treatment after kidney biopsy using a nationwide, large population-based data in Republic of Korea. We followed patients from 1 year before kidney biopsy and updated their exposure levels of glucocorticoid on a daily basis to allow for more accurate estimation of dose and duration of the exposure.

## Results

### Patient characteristics

**Figure 1:**
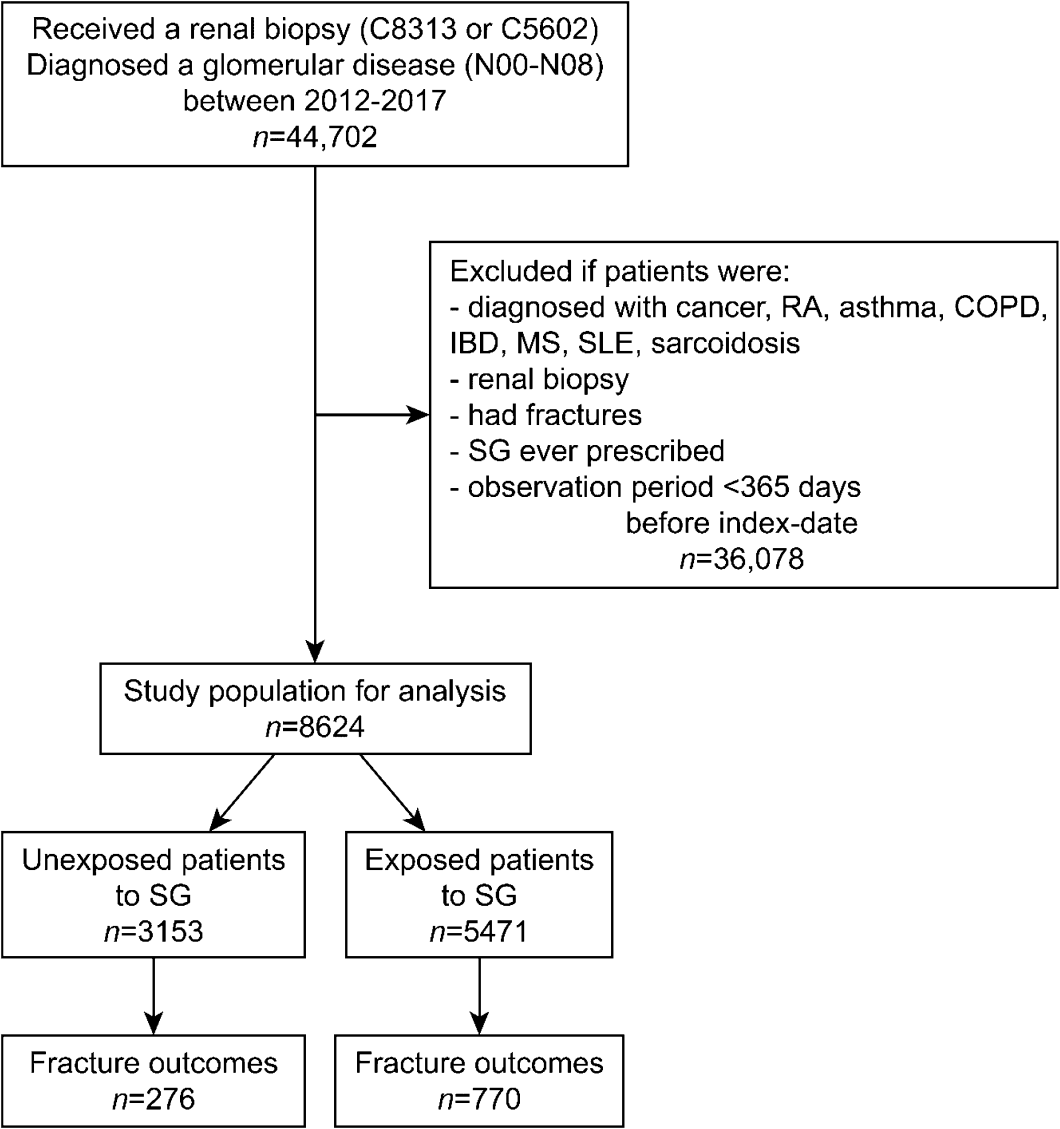
The flow chart of the study population. *RA* rheumatoid arthritis, *COPD* chronic obstructive pulmonary disease, *IBD* inflammatory bowel disease, *MS* multiple sclerosis, *SLE* systemic lupus erythematosus, *SG* systemic glucocorticoid.

A total of 44,702 patients were initially extracted from the HIRA database. Of them, 8,624 met all study inclusion criteria between January 2013 and December 2017 (Fig. 1). Among those patients who were diagnosed with glomerular disease, 5,471 (63.4%) were exposed to systemic glucocorticoids in the study period. Males were more prevalent in both groups (unexposed 64%, exposed 58%). The mean age at index date for the exposed group was 41 years old, older than that of the unexposed group. Exposed patients showed a more significant baseline comorbidity burden (based on the Charlson Comorbidity Index) and more frequent prescription history, including antidepressants and anti-osteoporosis medications than unexposed patients (Table 1). The mean (standard deviation) of the follow-up period for participants was 2.2 (1.5) years overall, 2.4 (1.4) for the exposed, 2.0 (1.4) for the unexposed. Of 8,624 patients in this analysis, 225 patients, 76 (2.4%) in the unexposed and 149 (2.7%) in the exposed, received dialysis after a renal biopsy based on a diagnosis code of ESRD and claim codes for dialysis. There was not a significant difference between them (p = 0.38).

**Table 1:** Demographic characteristics of the study population stratified by systemic glucocorticoid exposure during the pre-index period (1 year before a renal biopsy).

Characteristics	Steroid exposure	
	Unexposed (*n* = 3,153)	Exposed (*n* = 5,471)
**Sex, n (%)**		
Male	2031 (64)	3,200 (58)
Female	1,122 (36)	2,271 (42)
Age at index (year)	37 (17)	41 (18)
**Age group, n (%)**		
< 20	736 (23)	868 (16)
20–39	994 (32)	1605 (30)
40–60	1,084 (34)	2093 (38)
> 60	339 (11)	905 (17)
**Charlson comorbidity index condition, n (%)**		
0	1,245 (39)	1698 (31)
1	675 (21)	1,225 (22)
2	478 (15)	986 (18)
3 +	755 (24)	1562 (29)
**Concomitant medication, n (%)**		
0–5	812 (26)	974 (18)
6–15	1,192 (38)	1803 (33)
16–25	662 (21)	1,286 (24)
**> **25	487 (15)	1,408 (26)
**Selected medication, n (%)**		
Antiosteoporosis use^a^	79 (3)	304 (6)
Antidepressant use^b^	146 (5)	312 (6)

^a^Antiosteoporosis medications include alendronate, denosumab, fortical, ibandronate, miacalcin, raloxifene, risedronate, teriparatide, and zoledronic acid.

^b^Antidepressants include selective serotonin reuptake inhibitors, selective serotonin, norepinephrine reuptake inhibitors, serotonin modulators, tricyclics, other norepinephrine reuptake inhibitors, and monoamine oxidase inhibitors.

### Steroid exposure

We evaluated the level of systemic glucocorticoid exposure at various perspectives. Systemic glucocorticoid exposure for study populations was observed for an average of 33% of follow-up days. Of exposed patients, 38% had a daily dose of more than 20 mg/day. The majority of exposed patients received a cumulative dose of either less than 450 mg or greater than 3,600 mg (37%, 45%, respectively) in the study period. Exposed patients were prescribed a peak dose of greater than 60 mg/day most frequently. More than half (54%) of exposed patients had systemic glucocorticoids prescription days less than or equal to 120 days. Based on the Chi-square test, exposed patients were not equally proportionate to the level of systemic glucocorticoids prescription (P < 0.0001 for all, Table 2).

**Table 2:** Categorized glucocorticoid level of exposure variables among exposed patients during the study period.

Systemic GC exposure	Exposed (*n* = 5,471)	P-value
**Daily dose (mg/day)**		< 0.0001
≤ 5	344 (6)	
5 to ≤ 10	1,366 (25)	
10 to ≤ 20	1676 (31)	
> 20	2085 (38)	
**Cumulative dose (mg)**		< 0.0001
≤ 450	2044 (37)	
450 to ≤ 900	239 (4)	
900 to ≤ 1825	222 (4)	
1825 to ≤ 3,600	536 (10)	
> 3,600	2,430 (45)	
**Peak dose (mg/day)**		< 0.0001
≤ 10	896 (16)	
10 to ≤ 30	1,400 (26)	
30 to ≤ 60	1,133 (21)	
> 60	2042 (37)	
**Cumulative days**		< 0.0001
≤ 120	2,958 (54)	
120 to ≤ 180	424 (8)	
180 to ≤ 365	897 (16)	
> 365	1,192 (22)	

Values are presented as *n* (%). All glucocorticoid exposure variables were calculated with the equivalent dose (mg) according to the relative potency. The P-value calculated from the Chi-square test for goodness-of-fit is shown.

*GC* glucocorticoid.

### Fracture site and incidence

**Figure 2:**
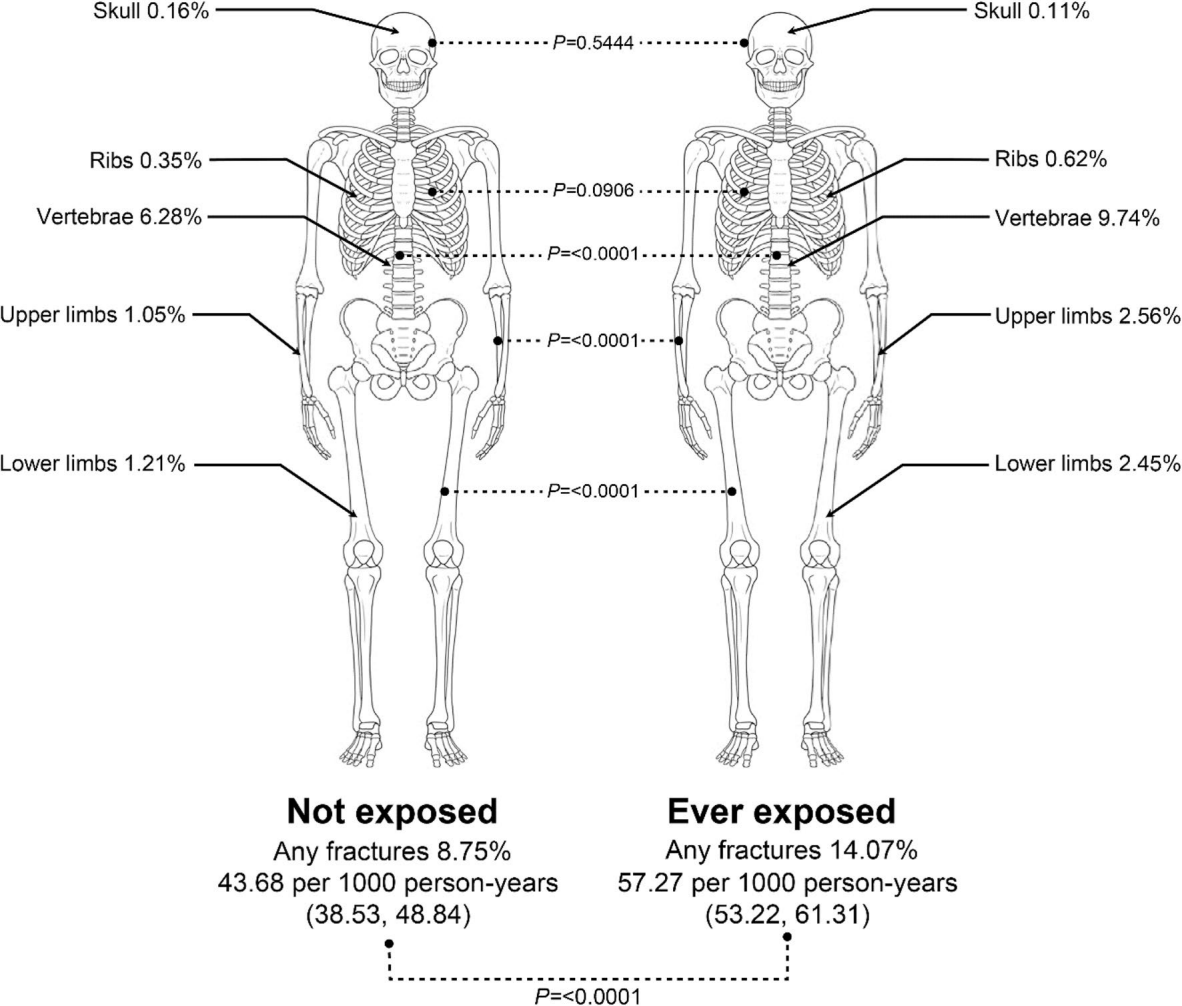
Comparison of fracture outcomes (including skull, ribs, vertebral, upper limb, and lower limb fractures) between the unexposed and exposed groups. The P-value calculated from the Chi-square test for independence is shown.

A total of 1,046 fractures of any kind were observed in the study period. We identified higher fracture incidence in the exposed group (unexposed 8.8% vs exposed 14.4%, P < 0.0001). Fracture incidences at different sites were compared between unexposed and exposed group. Fracture incidences at vertebrae, upper limb, and lower limb were greater in the exposed group than those in the unexposed group (P < 0.0001 for all, Fig. 2).

The fracture incidence rate was higher in the exposed group (5.7 per 100 person-year, 95% CI 5.32–6.13) than in the unexposed group (4.4 per 100 person-year, 95% CI 3.85–4.88). When we examined the fracture incidence rate by the level of systemic glucocorticoids exposure, early exposure to systemic glucocorticoids (≤ 120 days) showed the highest incidence rate (6.4 per 100 person-year, 95% CI 5.85–7.02, Table 3). The incidence did not show a dose dependent response.

**Table 3:** Crude fractures incidence rates (IRs) per 100 person-years by glucocorticoid level of exposure among all study populations (unexposed, *n* = 3,153; exposed, *n* = 5,471).

Systemic GC exposure	All fractures (*n* = 1,046)				
	*n*	%	Person-year	IRs	95% CI
**Steroid exposure**					
Unexposed	276	9	6,318.2	4.4	3.85–4.88
Exposed	770	14	13 445.6	5.7	5.32–6.13
**Daily dose (mg/day)**					
≤ 5	65	19	886.8	7.3	5.55–9.11
5 to ≤ 10	239	18	3,548.3	6.7	5.88–7.59
10 to ≤ 20	236	14	4,366.5	5.4	4.72–6.09
> 20	230	11	4,644.0	5.0	4.31–5.59
**Cumulative dose (mg)**					
≤ 450	361	18	5,351.8	6.7	6.05–7.44
450 to ≤ 900	33	14	532.3	6.2	4.08–8.31
900 to ≤ 1825	24	11	466.1	5.1	3.09–7.21
1825 to ≤ 3,600	59	11	1,139.7	5.2	3.86–6.50
> 3,600	293	12	5,955.8	4.9	4.36–5.48
**Peak dose (mg/day)**					
≤ 10	163	18	2,352.7	6.9	5.87–7.99
10 to ≤ 30	199	14	3,621.8	5.5	4.73–6.26
30 to ≤ 60	166	15	2,762.4	6.0	5.10–6.92
> 60	242	12	4,708.8	5.1	4.49–5.79
**Cumulative days**					
≤ 120	465	16	7,222.8	6.4	5.85–7.02
120 to ≤ 180	37	9	900.00	4.1	2.79–5.44
180 to ≤ 365	98	11	2021.2	4.8	3.89–5.81
> 365	170	14	3,301.6	5.1	4.38–5.92

*GC* glucocorticoid, *CI* confidence interval.

### Fracture risk

The fracture risk was further assessed by the time-dependent Cox regression model with exposed patients and the overall study population, respectively. Among the exposed group, the hazard ratio (HR) of fracture risk was the highest (HR 3.2, 95% CI 2.35–4.31) for patients who had a daily dose of 10–20 mg. The risk of fracture was higher in those having cumulative systemic glucocorticoid greater than 3,600 mg (HR 1.3, 95% CI 1.06–1.54) than in those having cumulative systematic glucocorticoid ≤ 450 mg.

In the overall study population, the exposed group had a remarkably higher HR of fracture risk (HR 6.0, 95% CI 5.01–7.23) than the unexposed group, indicating systemic glucocorticoid exposure was highly associated with fracture risks. The fracture risk for patients who had a daily dose of 5–20 mg was 3.8 times higher than the unexposed group. The HR of fracture risk for every level of systemic glucocorticoid exposure is shown in Table 4. The risk of fracture did not show a dose dependent pattern.

**Table 4:** Hazard ratio (HR) calculated from a time-dependent Cox model with each glucocorticoid level of exposure variable.

	Exposed (*n* = 5,471)		All patients (*n* = 8,624)			
	Model 1^a^		Model 2^b^		Model 3^c^	
	HR	95% CI	HR	95% CI	HR	95% CI
**Steroid exposure**						
Unexposed	–		Ref		Ref	
Exposed	–		6.0	5.01–7.23	5.9	4.88–7.06
**Daily dose (mg/day)**						
Unexposed	–		Ref		Ref	
≤ 5	Ref		2.7	2.01–3.56	2.6	2.00–3.46
5 to ≤ 10	2.4	1.82–3.21	3.8	3.18–4.59	3.7	3.10–4.48
10 to ≤ 20	3.2	2.35–4.31	3.8	3.11–4.54	3.6	3.01–4.40
> 20	1.0	0.70–1.38	1.6	1.27–1.95	1.6	1.25–1.92
**Cumulative dose (mg)**						
Unexposed	–		Ref		Ref	
≤ 450	Ref		2.0	1.72–2.36	2.0	1.69–2.32
450 to ≤ 900	1.4	0.95–1.93	2.4	1.65–3.40	2.2	1.55–3.21
900 to ≤ 1825	1.4	0.92–2.12	2.1	1.41–3.26	2.0	1.30–3.01
1825 to ≤ 3,600	1.3	1.01–1.80	2.0	1.49–2.66	1.9	1.39–2.48
> 3,600	1.3	1.06–1.54	2.0	1.66–2.38	1.9	1.59–2.29
**Peak dose (mg/day)**						
Unexposed	–		Ref		Ref	
≤ 10	Ref		2.4	1.98–2.93	2.4	1.96–2.90
10 to ≤ 30	1.6	1.32–2.02	2.8	2.32–3.39	2.7	2.25–3.30
30 to ≤ 60	2.7	1.86–3.00	3.4	2.74–4.12	3.2	2.62–3.94
> 60	1.1	0.85–1.41	1.8	1.47–2.22	1.8	1.43–2.15
**Cumulative days**						
Unexposed	–		Ref		Ref	
≤ 120	Ref		2.2	1.87–2.53	2.1	1.82–2.46
120 to ≤ 180	0.9	0.61–1.19	1.6	1.12–2.24	1.5	1.08–2.15
180 to ≤ 365	1.0	0.77–1.23	1.8	1.45–2.33	1.8	1.40–2.25
> 365	1.2	0.99–1.52	2.2	1.89–2.91	2.2	1.80–2.76

*CI* confidence interval, *Ref.* reference.

^a^Model 1 was with exposed patients only.

^b^Model 2 was with all study populations.

^c^Model 3 was adjusted for age group (≥ 60 years) and sex with all study populations.

We reanalyzed the fracture risk after adjusting for sex and older age (≥ 60 years) and found similar results. Older age, but not sex, was a significant factor in all analyses. Older age showed a significant effect on fracture risk (HR 1.2, 95% CI 1.05–1.44), even after adjusting for systemic glucocorticoid exposure. However, the history of prescribed antidepressants or anti-osteoporosis medications during the pre-index period was not associated with fracture risk (antidepressants: HR = 1.3, 95% CI 0.99–1.59; anti-osteoporosis: HR = 1.3, 95% CI 0.98–1.62).

## Discussion

In this nationwide population-based study for subjects who received kidney biopsy, we found that the risk of fracture significantly increased with glucocorticoid exposure. Majority of patients were males with a mean age of only around 40 years. Fractures occurred in 4.4/100 person-years in the unexposed group and in 5.7/100 person-years in the exposed group. The risk was significantly increased with increasing cumulative dose and age (≥ 60 years old). Most common fracture sites were vertebrae, upper limb, and lower limb in order. Incidence of fractures in all these sites was significantly higher in the exposed group than in the unexposed group. Fracture risk increased at all dose levels, including daily dose ≤ 5 mg/day.

Risk of fracture could be affected by independent associations with underlying diseases, disease activity, patient age, and sex. Although there exist evidences that glucocorticoid exposures can lead to increased fracture risk with a variety of inflammatory or immune-modulated diseases^9,10^, there are few studies on patients with kidney disease. Studies in a large-scale are especially rare. The main strength of our study was that we evaluated the risk of fractures associated with glucocorticoid use in selected patients who needed to receive kidney biopsy for their kidney disease. This study population was younger and more male-predominant than those of recent large-scale studies on the risk of fracture associated with glucocorticoid use in other inflammatory disease^2,5^. The cumulative dose of glucocorticoid used for kidney disease in this study was mostly 450 mg or less (37%) or 3,600 mg or more (45%). Cumulative days were also characteristic, with 54% of patients having less than 120 days and 22% patients having more than 365 days. A previous study of patients with RA has indicated significant dose–response trend of increasing fracture risk with increasing levels of exposure for both cumulative and daily doses^5^. Studies on RA and lung disease populations have also reported associations between cumulative dose and fracture risk and found that the cumulative dose is a stronger predictor of fracture than daily dose^11,12^. However, a prospective study on patients with RA, pulmonary, and skin disorders has shown that the risk of fracture is primarily related to the daily dose, not the cumulative dose^13^. Another study has reported that recent prolonged (≥ 3 months) steroid exposure, but not remote or short-term exposure, is associated with elevated fracture risk^14^. Although many studies have looked into the effect of glucocorticoids on fracture risk in patients with RA, there is no clear consensus about the magnitude of risk^15^. In the present study, the fracture risk increased at all dose levels, including daily dose ≤ 5 mg/day. Taken together, these results suggest that glucocorticoids can confer increased fracture risk even at a low daily dose and even short term exposure to glucocorticoid can increase the fracture risk. However, the risk of fracture was not dose and treatment duration dependent pattern. Some studies have reported that any steroid exposure poses a risk to bone health^2,9,16^. Glucocorticoid use increased the fracture risk in both young and old patients in this study, similar to findings of a previous study^2^. Using data of selected patients with kidney disease and a rigorous method for quantifying steroid exposure, our results extend previously published evidence, showing that glucocorticoid use could increase the risk of fracture. Our results suggest that management of glucocorticoid-induced fracture should be an important consideration in kidney disease care regardless of dose.

This study has several limitations that may confound the interpretation of its findings. First, it is subjected to limitations of observational research using claims-based data. Since identifying fracture cases based on diagnosis code solely is likely to cause misclassification problems due to potential miscoding, this approach can under- or over-estimate the real incidence rate of fractures. However, the Korean government regularly reviews NHIS to determine whether unfair claims are made based on wrong assessment. Second, the literature has also neglected to consider other impacts on the skeleton such as osteoporosis and intake of vitamin D supplements without doctor’s prescription, because those kinds of minor factor are not required to report as mandatory in South Korea. However, relation among osteoporosis, vitamin D supplements and fracture is controversial^13^. Third, there was no adjustment for the severity of the underlying kidney disease and dysfunction. It is possible that clinicians might have a long duration of glucocorticoids prescription for patients with low fracture risk or relatively tolerable status. In addition, kidney functions such as glomerular filtration rate and pathologic diagnosis after kidney biopsy were unavailable in this study. Thus, those with too advanced kidney dysfunction which could have mineral bone disorder or with too mild glomerular disease might have been excluded from glucocorticoid therapy. This selection bias may distort the truth of the increased fracture risk according to cumulative steroid dose. Despite these limitations, to the best of our knowledge, this is a study with the largest sample size to provide insights into fracture risk at different levels of glucocorticoid exposure among selected patients with kidney diseases for which glucocorticoids are commonly used in the therapy. Additional research is needed to better understand factors associated with increased bone fragility in those who are on glucocorticoids therapy. Future study should include clinical disease features (such as disease duration, activity, disease subtype, and treatment), environmental factors, and genetic factors.

In conclusion, the use of glucocorticoid is associated with an increased risk of fracture in kidney disease. This study supports the adage that age and use of glucocorticoid even at a low dose (less than 5 mg/day) with short term exposure could be important risk factors for glucocorticoid-induced fractures. Further research is warranted to understand the impact of glucocorticoid therapy in kidney disease and increased bone fragility on clinically relevant outcomes such as pain, physical activity, and quality of life.

## Methods

### Data source

All data were acquired from the Health Insurance Review and Assessment (HIRA) Service database, a Republic of Korean nationwide cohort set. Republic of Korea has the National Health Insurance System (NHIS), a public medical insurance system. HIRA conducts reviews and assessments of medical costs and quality services for the NHIS, including patient demographics (gender, age, and living area) and clinical data (diagnosis, procedures, and prescriptions)^17^. The NHIS provides mandatory health care for all Republic of Korean citizens, with an enrollment rate of 98%. The NHIS database has been previously used for epidemiological studies. Its validity has been proven in previous studies^18–21^. The HIRA collects claims data of 46 million patients (90% of the total population in Republic of Korea). The HIRA applies insurance codes for patients. These codes are needed to receive insurance coverage for relevant medical fees, resulting in a reliable method of disease identification from claims database.

### Ethical consideration

This study was approved by Ajou University Hospital Institutional Review Board (IRB number: AJIRB-MED-EXP-18-416). HIRA database was constructed after anonymization according to strict confidentiality guidelines. The requirement for informed consent was waived due to its retrospective nature. The attending government organization approved access to the HIRA Service for using the database (HIRA no. M20181212478).

### Study population

We selected patients who received a renal biopsy (C8313 or C5602) and had a diagnosis code of glomerular disease (N00–N08) according to the Korean Classification of Disease, seventh edition (KCD-7). The KCD code was developed for the compilation of health statistics and international comparison based on the International Classification of Disease 10. For each patient, the index date was the kidney biopsy date to set the beginning of follow-up. To follow patients up to 5 years and have a pre-index period of 12 months, data were extracted between January 1, 2012 and December 31, 2017, including the pre-index period. Patients were excluded if the following exclusion criteria were met during the pre-index period: (1) those were documented by relevant diagnosis codes such as cancer (C00–C97), RA (M79), asthma/chronic obstructive pulmonary disease (J44–J45), inflammatory bowel disease (K50–K52), multiple sclerosis (G35), lupus (M32, M60–M63), and sarcoidosis (D86); (2) those who had a renal biopsy; (3) those who had a diagnosis code of any fracture; (4) those who were prescribed with systemic glucocorticoid for any reason; and (5) those who had an observation period of less than 365 days. The steroid-exposed subjects of this study were those who received steroid prescriptions after a renal biopsy.

### Ascertainment of systemic glucocorticoid exposure

Systemic glucocorticoid exposures were carefully reviewed by the pharmaceutical main ingredient code from the HIRA drug prescription database. Patients were defined as being exposed to glucocorticoid if they had prescription claims with the main ingredient code of systemic glucocorticoid during the study period. Data on the prescription dose at one time, the number of times per day, and prescription days were collected to calculate total prescribed systemic glucocorticoid dosages. Only drug claims for oral, intravenous, and injected glucocorticoids were assessed, excluding intraarticular injections. Systemic glucocorticoid exposures were categorized by current daily dose, cumulative dose, cumulative exposure days, and peak dose. A daily dose implies the cumulative systemic glucocorticoid dose divided by cumulative exposure days, while a peak dose indicates the maximum daily dose per prescription. Cumulative exposure was not consecutive over available follow-up. We calculated the equivalent dose (mg) according to the relative potency of all systemic glucocorticoids and used it to assess steroid exposures. We defined cut-points for the level of glucocorticoid exposure based on taking 5 mg prednisone. Specifically, cut-points for a cumulative dose of glucocorticoid for 3 months, 6 months, 1 year, and 2 years of 5 mg prednisone per day were 450, 900, 1825 and 3,600 mg, respectively.

### Ascertainment of fracture

All claims records of study patients from the NHIS database were utilized to identify fracture incidence by relevant KCD codes. In addition to diagnosis codes, fractures required associated outpatient visits or hospital admissions from Orthopedics, Neurosurgery, Department of Anesthesiology and pain medicine, and Rehabilitation Medicine. The primary outcome of this analysis was the fracture incidence, including hip, radius/ulna, pelvis, humerus, femur, vertebral fractures, and others. The event date of fracture was the earliest diagnosis date of the fracture.

### Statistical methods

Patient characteristics during the pre-index period were summarized by systemic glucocorticoid exposure. We investigated various level of systemic glucocorticoid exposure using Chi-square test of goodness-of-fit to see whether data were equally distributed into intervals. We also compared the incidence of overall fracture as well as specific factures (hip, radius/ulna, pelvis, humerus, femur, and vertebral fractures) between the exposed group and unexposed group using Chi-square test. We calculated the fracture incidence rate per 100 person-years to assess the risk of fracture in an individual subject over a specified period. Incidence rates per 100 person-years were also stratified by glucocorticoid exposure levels. For each patient, follow-up for fracture outcomes began at the index date and extended through the earliest censoring event (fracture incidence, diagnosis of cancer, the end of observation date, i.e., December 31, 2017).

Further, we fitted a Cox proportional hazard model to assess the effect of covariates on the risk of fracture. However, the proportional hazard assumption was not met. The interaction of time and covariates was significant. Therefore, we incorporated changes over time into this analysis by using a modified Cox model, a time-dependent Cox regression model, for which we modified the time to the event, log-transformed it, and then subtracted its mean. We performed this analysis for the exposed group and the overall study population separately. We then reanalyzed this model by controlling for confounding effects of older age (≥ 60 years) and sex. The effects of antidepressant medication and anti-osteoporosis medication during the pre-index period were also investigated. All analyses were performed using SAS software, version 9.4 (SAS Institute, Cary, NC, USA). A P-value < 0.05 was considered statistically significant.

### Ethical approval

All procedures performed in studies involving human participants were in accordance with the ethical standards of the institutional and/or national research committee and with the 1964 Helsinki declaration and its later amendments or comparable ethical standards.

### Informed consent

For this type of study formal consent is not required.
